# Supplementary material for: Knowledge of behavioral risk factors for type 2 diabetes mellitus and its associated factors among women of reproductive age
Source: PLoS One. 2025 Feb 13;20(2):e0275700. doi: 10.1371/journal.pone.0275700 (PMC11824971; doi:10.1371/journal.pone.0275700)
Supplement: Attachment — Submitted filename: Response to Reviewers DOC.docx [file pone.0275700.s001.docx]

**RESPONSE TO REVIEWER**

[Cover Letter]

Dear Editor,

We appreciate you and the reviewers' precious time in reviewing our paper and

providing valuable comments. It was your worth and insightful comments that led to

possible improvements in the current version. The authors have carefully considered the

comments and tried our best to address every one of them. We hope the manuscript after

Your careful revisions meet your high standards. The authors welcome further constructive

comments, if any.

Below, we provide the point-by-point responses. All modifications to the manuscript have

been highlighted in blue on a white background.

Sincerely,

Tinsae Seyoum

tswy2006@gmail.com

lecturer, Department of Public Health

Arba Minch College of Health Sciences

**[General comments]** ‘’When submitting your revision, we need you to address these additional requirements.

1. Please ensure that your manuscript meets PLOS ONE's style requirements, including those for file naming.

2.You indicated that you had ethical approval for your study. In your Methods section, please ensure you have also stated whether you obtained consent from parents or guardians of the minors (participants below the age of 18 years) included in the study or whether the research ethics committee or IRB specifically waived the need for their consent.

3. When completing the data availability statement of the submission form, you indicated that you will make your data available on acceptance. We strongly recommend all authors decide on a data sharing plan before acceptance, as the process can be lengthy and hold up publication timelines. Please note that, though access restrictions are acceptable now, your entire data will need to be made freely accessible if your manuscript is accepted for publication. This policy applies to all data except where public deposition would breach compliance with the protocol approved by your research ethics board. If you are unable to adhere to our open data policy, please kindly revise your statement to explain your reasoning and we will seek the editor's input on an exemption. Please be assured that, once you have provided your new statement, the assessment of your exemption will not hold up the peer review process.

4. Please include your tables as part of your main manuscript and remove the individual files. Please note that supplementary tables (should remain/ be uploaded) as separate "supporting information" files

**Response** : Thank you for your remembrance about worthy comments on important issues regarding the manuscript preparation and statements that need to addressed.

This manuscript is prepared according to the PLOS guideline.

We stated the following idea of the method section about consent and assent on Page 6, lines 151–153:After obtaining consent from respondents whose age is above 18 years and from parents and guardians for those minority participants, they were included in the study.'

Concerning data availability, we declared that the entire data is freely accessible if the manuscript is accepted for publication.
Concerning the table, we included all tables in the main manuscript and removed the file that was uploaded previously.

**[General Comment]** ‘’This is an important article on knowledge of behavioral risk factors of

Diabetes among reproductive age group women in Ethiopia. However, the manuscript needs

editing throughout in terms of English language, typos, abbreviations and the result section needs to be articulated in a proper fashion. Thanks’’

**Response to Reviewer 1**

.

.

**Response:** Thanks for your valuable comments. We revised the English language and typos and abbreviations that needed clarification, we and tried to articulate the result section in the proper way as much as possible.

The result section also revised from page 8-16.

**Response to Reviewer 2**

**[General Comment]** ‘’I was kindly asked to review the manuscript of Seyoum T et al. I

thank the authors for their work, as the result is interesting and achieved through a very

well described and reproducible methodological rigor. I believe the article may also be of

interest to readers of the journal, but before publication, it should, in my opinion, be fixed

in form, following the suggestions given below.’’

**Response:** Thank you very much for your comments that helped us improve this manuscript.

**[Comment 1] ‘’**Editing for English language is recommended.

Line numbers in the manuscript file are missing.’’

**Response:** Thanks for your kind reminders. We edited almost all sentences thoroughly and provided line numbers in the manuscript file.

**[Comment 2]** Title:

‘’I suggest deleting “2022” at the end of the title and using “…in Arba Minch, Gamo

zone, Southern Ethiopia” here and everywhere into the main text, abstract included.’’

**Response:** Thanks. We edited the title page, abstract and the main texts by removing ‘’2022’’ and’’ in Arba Minch, Gamo zone, South Ethiopia’’ except from the tables and figures.

**The revised Title**: Knowledge of behavioral risk factors for type 2 diabetes mellitus and its associated factors among reproductive-age women on page 1, line1-2

Abstract: **Objectives**, To assess knowledge of the behavioral risks of type 2 diabetes mellitus and its associated factors among women of reproductive age./page 2,line 21-22

**[Comment 3] ‘’**Corresponding author:

Please, report on the same line the corresponding author and the email address’’

**Response:** Thanks for your kind reminders. We edited accordingly.

*= Corresponding author: Email [address, tswy2006@gmail.com](mailto:address,tswy2006@gmail.com) page no1,line 6

**[Comment 4] ‘’**Authorship:

Please, verify with the Editor that, beside a first joint authorship, another equal

contribution is admitted.

Please, delete the #aArba Minch, South Nation Nationality Region, Ethiopia, as

affiliations report this information.’’

**Response:**Thanks a lot for your comment. We verified the equal contribution of the authors.

We removed the’ #a Arba Minch South Nation Nationality Region, Ethiopia’ from cover page 1.

**[Comment 5]** ‘’Abstract, page 2:

Define abbreviations upon first appearance in the text. For example, the abbreviations AOR,

BRF and DM are cited, but not defined.

Please, verify the order of words at the end of the Objective sentence and as suggested

previously.’’

**Response:** Thank you for your nice suggestion. We revised the abbreviation on the abstract page:“Adjusted Odd Ratio, Behavioral Risk Factors, Diabetes Mellitus”

The objective was also amended according to your suggestions and comments by removing ‘2022’ and’ in Arba Minch town, South Ethiopia’

**[Comment 6]** ‘’Introduction:

Line 2, please add a space between diabetes and (T2DM)

Line 28, please remove the space at the beginning of the sentence.

Line 32, please define the abbreviation “RA” before using it

Line 35-38, please evaluate to move this paragraph before the sentence “As a result, …” at line

31 or in the discussions section.’’

**Response:** Thank you very much for the reminder. We have made revisions accordingly.
We add space between T2 and DM with a hyphen (T2-DM).
We totally removed RA and defined the full word with reproductive age. 
We moved the paragraph 'Knowledge of BRF is a prerequisite for preventing diabetes in reproductive age groups’ into the discussion session. Page 17, lines 321–322

**[Comment 7]** ‘’Results:

I suggest renaming sub-headings or numbering the entire section as “5.RESULTS” and the ‘’

**Response:** Thank you for the nice reminder. We renamed the subheading section and removed numbering styles.

**[Comment 8]** ‘’Materials and methods:

Please, define the study design: qualitative, quantitative or mix-method?

Please, correct the study period: from February 20, 2022 to March 22, 2022, not

February20/2022.

Please, correct “women of reproductive age”. In my personal opinion, a woman of

reproductive age is 46 years old, at maximum. I strongly recommend to define the fertile

age among inclusion criteria, exclude from the study women of more than 46 years old and

revise the manuscript accordingly.

What about parity? It should be of interest to analyse wherever parity >0 may be associated

with higher or lower consciousness.’’

**[Comment 9]** ‘’Discussion and Conclusion:

**Response:** Thank you for your comments and we revised accordingly as follow:

We defined the study design by adding ''quantitative'' on page 4, line 95.
We corrected the study period from February 20, 2022, to March 22, 2022. (page 4, lines 95–96)
We changed the text to women of reproductive age” in all our text, page 1, lines 2–3, lines 19, 22, and also on the key part.
Concerning participants’ age, we used the WHO and Ethiopian Demography Survey to consider that a woman of reproductive age is up to 49 years older than 46, and all analyses were made by age between 15 and 49. Otherwise, if we consider up to 46, it could affect the whole data (its result). Hence, we explained on page 4, lines 99–100.
Unfortunately, in this current study, we missed the ''parity’’ variable, so we recommended that the future be addressed in the recommendation part. (page 20, line no. 404).

Section are numbered. Either all sections are numbered, or no section is numbered, your choice!’’

**Response:** Thank you for your nice reminder. We revised by removing numbers from all

Sections.

**[Comment 10] ‘’**References:

Please, use proper formatting and the same font.

What does it mean “uncategorized references”?’’

**Response:** Thanks for your suggestion.

We revised the fonts in the reference to be the same at all levels and adjusted the formats accordingly, and the uncategorized part was totally removed .

**Response to Reviewer 3**

**[Comment 1]”** Introduction

The manuscript didn’t give a clear idea of the central question that the research is

intended to answer and its justification.

Make sure you provided a sufficient detail on the magnitude of T2DM among reproductive-age women and the consequences for those affected

The outcome of interest “Knowledge of BRF for T2DM” should have been the central idea in a discussion of why certain factors need more investigation if the problem is to be fully understood. Rather it was "BRF-T2DM" " "behavioral changes -Knowledge" BRF Knowledge - T2DM prevention"…

The argument on non-existence of studies on this topic in Ethiopia was inconsistent with the fact that numerous studies were used to compare and contrast findings later on the discussion section.

The statement on significance of the study doesn't identify the beneficiaries and the benefits of this specific study clearly.’’

**Response:** Thank you very much for pointing this out.

To provide sufficient detail on magnitude, we add an idea and citation on page 3. Lines 54–59: ‘’Women with diabetes are more likely to develop cardiovascular disease, and the risk of developing heart disease is approximately four times higher than that of men (8). In women, diabetes is one of the leading causes of death (9, 10). The number of cases will also be expected to rise to 222 million by 2030, and nearly 2.1 million women will die of diabetes; moreover, in the next two decades, the greatest number of females will be expected, and of those, more than 90% of the contribution will be from our continents (11).’’

We revised the sentence to explain why this study needed it as follows: [In page 4, lines 77–81], we rearrange the sentence and add, ‘Knowledge of BRF is also a prerequisite for preventing DM in reproductive age groups. Previous studies revealed more about the knowledge of some general risk factors (sedentary life,obesity,poor diet, and not getting enough exercise) among diabetes and chronic patients but limited the rest of the behavioural risks in WRA (14, 9–26). In Ethiopia, more studies have also focused on general knowledge of DM, and there is also limited evidence on women of reproductive age's knowledge of BRFs for T2-DM. (27–29).

We also revised and rewrote the study benefit and beneficiaries on page 4, lines 82–91, as follows: “Understanding diabetes risk factors in the population across diverse socio-demographic and economic categories will lead to better preventative behaviours, which will benefit future studies. The WRA group will benefit primarily from this study. If they are informed about the risk factors, they will be less likely to engage in risky behaviors.

Therefore, the aim of this study is to assess the knowledge of all possible BRFs towards T2-DM and its associated factors. The study's findings will help determine the level of knowledge regarding the BRF of T2-DM among WRA and also identify the significant association with it. It might also offer baseline information for intervention programmes that support increasing knowledge on healthy lifestyle modification and prevention in the future. In addition to this, it will also serve as a reference for other scholars.”

**[Comment 2] ‘’**Materials and Methods

The manuscript contain errors in the design and conduct of research.

For example, the statement on Sample size determination was incorrect given their statistical implications. “q =proportion of people”’’

**Response:** Thanks for your comment. We removed the q= proportion of people,

Some text modification was made to be more coherent on page 5, lines 111-113

**[Comment 3] ‘’**A multi-stage sampling strategy doesn’t provide sufficient details on the sampling units and sample allocation and selection procedure.’’

**[Comment 4]** ‘’Variable of the study doesn't identify all the variables used in the analysis including Awareness on DM, Knowledge of Type-2DM, Knowledge of symptoms of Type 2-DM, Knowledge relate to prevention of DM and its complication, Behavioral risk Perception for Type 2DM, Adopting healthy life style for Type 2 DM.’’

**Response:** Thanks a lot for these comments. We revised the manuscript and elaborated with detail on sampling units and sample selection procedures with modification on page no 5, lines 116-125 as follows: ‘using a systematic random sample approach, the study unit was chosen among households. The first house was selected by the lottery method, and then the next house was selected after calculating the sampling interval (k = 13). Then, every 13^th^ house was taken until the required sample size was fulfilled. If more than one individual meets the inclusion criteria in the household, one participant would be selected using the lottery method. Then, for those randomly selected reproductive-age women, a face-to-face interview was done.’

**Response:**Thanks for your reminder. We added the variable that was identified in the analysis. We included awareness of DM, knowledge of T2-DM, knowledge of symptoms and knowledge related to prevention of DM and its complication,behavioral risk perception for type 2 DM and adopting a healthy life style in our analysis but all of them were not significant.p 5, lines129-131.

**[Comment 5]** ‘’Data collection was incorrect given their statistical implications “a pre-test of 5 % of the sample population out of the study area” and also described repeatedly. “after sampling technique” and “under a section titled data collection”

Data processing and analysis was incorrect with regard to description of Statistical program used and their purpose. “Epi Data 4.6” Statistical procedures used to modify raw data before analysis. “Combining wrong or uncertain responses in determining level of knowledge of the behavioral risk of T2DM”

Variables used in the analysis “demographic factors” and “the level of knowledge of the behavioral risk of T2DM”

Summary statistics and graphical techniques used in descriptive statistics for each variables.

Methods used for analysis coherently. “Binary logistic regression analysis” “multivariable logistic regression” “Descriptive statistic”

Purpose and reporting format to be followed for the analysis made “Binary logistic regression analysis to identify determinants of behavioural risk factors for T2DM… crude odds ratio (COR) and adjusted odds ratio (AOR) with its respective 95% confidence interval (CI) was used to the number of measurement stations included. interpret the result. Then, multivariable logistic…”

**Response:** Thank you for the nice reminder. To provide a statistical impression of the text, we readjusted the statements as follows: 
"A pretest was conducted with 5% of the sample population who lived outside the study area (Mirab Abaya town)’’, and the word that was described repeatedly." was readjusted in the section entitled Data Collection, page 6, lines 145–147, and the repetition of the statement that tells about the pretest in the sampling was totally removed. 
Regarding the description of the statistical programme used and its purpose, it was restated as follows: on page 6, lines 163. ’Epi Data 4.6 version was used for data entry, and SPSS version 21 was used for all statistical analyses.’
Statistical procedures are used to modify raw data before analysis. “Combining wrong or uncertain responses in determining the level of knowledge of the behavioural risk of T2DM” is removed
Variables used in the analysis are also described as follows, with a restatement on page 7, lines 170-172: ‘A descriptive statistic was used to describe the study participants in relation to relevant variables.’
In order to make the sentence coherent, we restructured it as follows on page 7, lines 171-178: ’ A binary logistic regression analysis was conducted to identify the determinants of knowledge and assess the association between the independent and dependent variables. The crude odds ratio (COR), 95% confidence intervals (CI), and P-values were used to interpret the result. Variables with p-values less than 0.25 during bivariate analysis were transferred to multivariate analysis to control for confounding effects. Then, multivariate logistic regression was used to control the possible confounders with an adjusted odds ratio (AOR) of 95% CI and p-value. Those variables with a p-value of < 0.05 in the multi-variable analysis were considered significantly associated with the outcome variable.’

[**Comment 6**] ‘’Results

The manuscript contain errors in the application, analysis, interpretation, and reporting of

statistics. For example

Age was with different measures that requires difference in properties of distribution. “

minimum age of respondents is 15-year-old and maximum age is 49 years” and “mean age of respondents was 27.3 (SD+8.2 years).”

Continuous variable was also reported without measures of variance. “Average family income was 3910.00 Eth birr(--- SD)”

Inconsistent format was used in reporting distribution of variables. 235 (37.7%) were collage and above, “married (57.6%)”, “Half of the participants (312, 50.1%) knew T2DM”

The interpretation of results disregard group sizes for each analysis in many instances in the manuscript. “Source of information (Health workers -116)”, “Have got DM health education (Yes- 159)”

The text report of the regression analysis states a measure of precision (a confidence interval and P values) for each explanatory variable which is unnecessary.

Erroneous interpretation of result was made in some instances. “A household with an average income of more than 5,001 Eth. Birr was also 1.93 times...”

There is no report of whether the variables were assessed for interaction.’’

**Response:** Thanks for your comment. We changed the interpretation of the result and focused on the statistical impression.

- The result section was revised according to the comments (pages 8–16).
  Regarding the age, it was written as follows: the minimum age of respondents is 15 years old, and the maximum age is 49 years old. The mean age of respondents was 27.3 (SD + 8.2). (page 8, lines 197).
  The continuous variable is rewritten with SD as follows: the average family income per month was 3910.00 Eth Birr (SD + 3107.91) under the result section. (page 8, line 201-202).
- We also arranged and revised the format we used for the number and percent in brackets throughout the paper: Concerning educational status, those who were illiterate and able to write and read were 27 (4.3%), respectively, and 235 (37.7%) participants were educated in college or above on page 8, lines 198–200.
- Regarding awareness, almost 568 (91.2%) of study participants had heard about DM, and 264 (42.2%) had gained information from friends or relatives. From the total study participants, 159 (25.4%) had received health education about DM, and those who had friends or relatives with diabetes were 192 (31%) on page 9, lines 207–209.
  Concerning the interpretation of the source of information, those who get health education from health workers in the health facilities (direct face-to-face) were 159.
  Source of information for the first time who get 116
- We restructured the table accordingly. The text report of the regression analysis that stated a measure of precision (a confidence interval and P values) for each explanatory variable was removed from all tables and considered on AOR only in multivariate and bi-variate analyses. Hence, we removed the P-value from the COR column (pages 15–16).
  The interpretation of the result on average family income was also revised as follows: The average monthly family income of between 3000 and 5000 Eth. Birr was 1.81 times more likely (AOR = 1.81, 95% CI = 1.03–3.18) to have good knowledge about BRF than those with a monthly family income of less than 1,999 Eth. Birr, and among respondents with an average monthly family income of more than 5,001 Eth. Birr, it was also 1.93 times more likely (AOR = 1.93, 95% CI = 1.02–3.68)  to have good knowledge about BRF than its counterparts’ on page 15, lines 291–296.
- We also assessed the interaction between variables and explained it as follows: The assumption of multicollinearity was checked, and there is no multicollinearity among the independent variables. This assumption was tested using the variance inflation factor (VIF) values on page 15, lines 306-307.

**[Comment 7]** ‘’Discussion

The discussion was unjustified, inappropriate, and erroneous in many instances in the

manuscript. “The knowledge gap in the WRA may worsen the burden of the illness.”

The discussion was also made for each explanatory variable which fails to show

association with outcome variable unnecessarily.’’

**Response:** Thanks for your comment. We agreed with you that the discussion on the generalized sentence (“The knowledge gap in the WRA may worsen the burden of the illness.”) modified into ‘The knowledge gap in the WRA may worsen the burden of the BRF of T2-DM’. page 17, lines 324.

The discussion that was explained about variables that fail to show association was completely removed from pages 16-17.

**[Comment 8]** ‘’Conclusions

Conclusions are presented in inappropriate way. “Nearly 60% of the respondents had no

idea about others T2DM risk factors”

The conclusions was also overstated and erroneously discussed possible implications of the results out of the context of data presented in the manuscript. “This might indicate

that women are ignoring their health.”

The conclusions didn’t provide sufficient details on the association of interest that is “

BRF Knowledge” and “Friend /relatives” “Family” “Health worker”’’

**Response:** Thanks for the critical comment. We deleted unnecessary words and modified conclusion part as follows:on page 19,line no 364-377.

Overall, compared with the previous study regarding the BRF of T2-DM, the level of knowledge was less than half of the participants (47%). There was a statistically significant association between average family incomes of greater than 3000 Eth.Birr, family history of diabetes, DM in a friend or relative and sources of information from health workers, friends and relatives,which had a more likely strong association with the level of BRF’s knowledge.

A poor score on knowledge could indicate a knowledge gap. Women, in particular the reproductive age group, need to have an in-depth understanding of BRF in order to improve their health and prevent the consequences.

To better manage, all women demand equal access to information. Consequently, knowledge could aid in determining diabetes’s modifiable risk. A community-based education programme about T2-DM risk factors should also be conducted among the people at regular intervals so as to increase their knowledge.

To summarize, detailed education on diabetes and socio-demographic and economic factors  also needs to be observed in order to improve their understanding of T2-DM risk factors.

**[Comment 9] ‘’**General comments

The language in this manuscript was difficult to understand in many instances and includes.

Grammatical error: for instance ".. is rises.."

Typographical error such as "from he study period"

Unconventional and inappropriate use of abbreviations "RA"

Issues with substantial clinical implications for example " T2DM is evolving"

- **Response:** Thanks for the comment. We revised accordingly and also removed unconventional abbreviations, which were corrected by defining the word carefully (women of reproductive age). Page 4, lines 80-81
- The incidence rises on Page 3, Lines 61-62.
- ‘T2DM is evolving’ change to T2-DM is developing on page 3, line 60.
- The text was also modified to be coherent and fluent on page 3, lines 70–74, as follows:’There are several predisposing factors for T2-DM: Broadly classified into non-modifiable (family history of diabetes and age) and modifiable risk factors. The BRFs are parts of modifiable factors that can increase the chance of developing T2-DM; they include obesity, physical inactivity, consuming an unhealthy diet, heavy alcohol and tobacco use, sleeplessness, and stress (10, 11). These problems are hidden in low-socioeconomic-status countries, including our country (12, 13).’

**[Comment 10]** ‘’In conclusion

These errors are serious enough to question the conclusions.

I recommend authors to read PLOS ONE manuscript submission guidelines and seek editorial help in research report write-up.

Please refer to the revised manuscript.’’

**Response:** Thank you very much for your valuable comment. We modified the point that needed improvement and rewrote the research report based on the submission guidelines.
