# Supplementary material for: Knowledge of behavioral risk factors for type 2 diabetes mellitus and its associated factors among women of reproductive age
Source: PLoS One. 2025 Feb 13;20(2):e0275700. doi: 10.1371/journal.pone.0275700 (PMC11824971; doi:10.1371/journal.pone.0275700)
Supplement: Attachment — Submitted filename: Response to Reviewers of PLOSE.docx [file pone.0275700.s002.docx]

**RESPONSE TO REVIEWER**

[**Cover Letter**]

Dear Editor,

We genuinely appreciate you and the reviewers for taking the time to review our paper and provide valuable comments. Your insightful feedback has significantly contributed to the improvements in the current version. We have carefully considered each comment and have made every effort to address them. We hope that the revised manuscript meets your high standards. We also welcome any further constructive comments. Below, we provide point-by-point responses. All modifications in the manuscript have been highlighted in blue on a white background.

Sincerely,

Tinsae Seyoum (PI)

[tswy2006@gmail.com](mailto:tswy2006@gmail.com)

+251913260781

Lecturer, Department of Public Health

Arba Minch College of Health Sciences

**[General comments]** ‘’When submitting your revision, we need you to address these additional requirements.

1. Please ensure that your manuscript meets PLOS ONE's style requirements, including those for file naming. The PLOS ONE style templates can be found at
   [https://journals.plos.org/plosone/s/file?id=wjVg/PLOSOne_formatting_sample_main_body. pdf](https://journals.plos.org/plosone/s/file?id=wjVg/PLOSOne_formatting_sample_main_body.%20pdf) and [https://journals.plos.org/plosone/s/file?id=ba62/PLOSOne_formatting_sample_title_authors_affiliations.pdf](https://journals.plos.org/plosone/s/file?id=ba62/PLOSOne_formatting_sample_title_authors_affiliations.pdf#inbox/_blank)

2. You indicated that you had ethical approval for your study. In your Methods section, please ensure you have also stated whether you obtained consent from parents or guardians of the minors (participants below the age of 18 years) included in the study or whether the research ethics committee or IRB specifically waived the need for their consent.

3. When completing the data availability statement of the submission form, you indicated that you will make your data available on acceptance. We strongly recommend all authors decide on a data sharing plan before acceptance, as the process can be lengthy and hold up publication timelines. Please note that, though access restrictions are acceptable now, your entire data will need to be made freely accessible if your manuscript is accepted for publication. This policy applies to all data except where public deposition would breach compliance with the protocol approved by your research ethics board. If you are unable to adhere to our open data policy, please kindly revise your statement to explain your reasoning and we will seek the editor's input on an exemption. Please be assured that, once you have provided your new statement, the assessment of your exemption will not hold up the peer review process.

4. Please include your tables as part of your main manuscript and remove the individual files. Please note that supplementary tables (should remain/ be uploaded) as separate "supporting information" files

**Response** :

**[General Comment]** ‘’This is an important article on knowledge of behavioral risk factors of **Diabetes** among reproductive age group women in Ethiopia. However, the manuscript needs editing throughout in terms of English language, typos, abbreviations and the result section needs to be articulated in a proper fashion. Thanks’’

**Response to Reviewer 1**

.

.

**Response:** Thanks for your valuable comments. We revised the English language and typos and abbreviations that needed clarification. We tried to articulate the result section in the proper way as much as possible for the entire paper (from the abstract to the conclusion page).

**Response to Reviewer 2**

**[General Comment]** ‘’I was kindly asked to review the manuscript of Seyoum T et al. I thank the authors for their work, as the result is interesting and achieved through a very well described and reproducible methodological rigor. I believe the article may also be of interest to readers of the journal, but before publication, it should, in my opinion, be fixed in form, following the suggestions given below.’’

**Response:** Thank you very much for your comments that helped us improves this manuscript.

**[Comment 1] ‘’**Editing for English language is recommended. Line numbers in the manuscript file are missing.’’

**Response:** Thanks for your kind reminders. We edited almost all sentences thoroughly and provided line numbers in the whole manuscript file.

**[Comment 2]** Title:

‘’I suggest deleting “2022” at the end of the title and using “…in Arba Minch, Gamo zone, Southern Ethiopia” here and everywhere into the main text, abstract included.’’

**Response:** Thanks. We edited the title page, abstract and the main texts by removing ‘’2022’’ and’’ in Arba Minch, Gamo zone, South Ethiopia’’ except from the tables and figures.

**The revised Title**: Knowledge of behavioral risk factors for type 2 ddiabetes mellitus and its associated factors among reproductive-age women on page 1, line1-3

Abstract: **Objectives**, To assess knowledge of the behavioral risks of type 2 diabetes mellitus and its associated factors among women of reproductive age./page 2, line 22-23/

**[Comment 3] ‘’**Corresponding author:

Please, report on the same line the corresponding author and the email address’’

**Response:** Thanks for your kind reminders. We edited accordingly.

*= Corresponding author: Email [address, tswy2006@gmail.com](mailto:address,tswy2006@gmail.com) page no1, line 6

**[Comment 4] ‘’**Authorship:

Please, verify with the Editor that, beside a first joint authorship, another equal contribution is admitted.

Please, delete the #aArba Minch, South Nation Nationality Region, Ethiopia, as affiliations report this information.’’

**Response:** Thanks a lot for your comment. We verified the equal contribution of the authors.

We removed this symbol'#@' and added these symbols**^¶,^** ^&^ on equal contribution based on PLOSE rule.

We removed the sentence '#a, Arba Minch, South Nation Nationality Region, Ethiopia.' from cover page.(Page 1)

**[Comment 5]** ‘’Abstract, page 2:

Define abbreviations upon first appearance in the text. For example, the abbreviations AOR,

BRF and DM are cited, but not defined.

Please, verify the order of words at the end of the Objective sentence and as suggested previously.’’

**Response:** Thank you for your nice suggestion. We revised the abbreviation on the abstract page2, line no. 30, 31, 32, and 39:“Adjusted Odd Ratio, Behavioral Risk Factors, and Diabetes Mellitus”

The objective was also amended according to your suggestions and comments by removing ‘2022’ and’ in Arba Minch town, South Ethiopia’

**[Comment 6]** ‘’Introduction:

Line 2, please add a space between diabetes and (T2DM)

Line 28, please remove the space at the beginning of the sentence.

Line 32, please define the abbreviation “RA” before using it

Line 35-38, please evaluate to move this paragraph before the sentence “As a result, …” at line

31 or in the discussions section.’’

**Response:** Thank you very much for the reminder. We have made revisions accordingly.

We detached the paragraph of ‘Knowledge of BRF is also a prerequisite for preventing diabetes in reproductive age groups’into the discussion session.

**[Comment 7]** ‘’Results:

I suggest renaming sub-headings or numbering the entire section as “5.RESULTS” and the ‘’

**Response:** Thank you for the nice reminder. We renamed the subheading section and removed numbering styles.

**[Comment 8]** ‘’Materials and methods:

Please, define the study design: qualitative, quantitative or mix-method?

Please, correct the study period: from February 20, 2022 to March 22, 2022, not February20/2022.

Please, correct “women of reproductive age”. In my personal opinion, a woman of reproductive age is 46 years old, at maximum. I strongly recommend defining the fertile age among inclusion criteria, excluding from the study women of more than 46 years old and revising the manuscript accordingly.

What about parity? It should be of interest to analyses wherever parity > 0 may be associated with higher or lower consciousness.’’

**[Comment 9]** ‘’Discussion and Conclusion:

**Response:** Thank you for your eminent comments and we revised accordingly as follow:

We defined the study design by adding ''quantitative'' on page 4, line no. 91

We corrected the study period from February 20, 2022, to March 22, 2022. (Page 4, line no.92)

We changed the text to “women of reproductive age” in all our text, page1, line 2-3; page 2, lines no 20, 23, 31and line 43 including key words.

Concerning participants’ age, we relied on the data of the WHO and Ethiopian Demography Health Survey (EDHS) to consider that a woman of reproductive age is up to 49 years older than 46, and all analyses were made by age between 15 and 49. Otherwise, if we consider the age up to 46, it could affect the whole data and its result.

Unfortunately, in this study, we didn't consider the ''**parity**’’ variable, so we recommended for future to be addressed in the recommendation part. (Page 19, line no 388-389).

Section are numbered. Either all sections are numbered, or no section is numbered, your choice!’’

**Response:** Thank you for your nice reminder. We revised by removing numbers from all

Sections

**[Comment 10] ‘’**References:

Please, use proper formatting and the same font.

What does it mean “uncategorized references”?’’

**Response:** Thanks for your suggestion.

We revised the fonts in the reference to be the same at all levels and adjusted the formats by using endnote accordingly, and the uncategorized part was totally removed.

**Response to Reviewer 3**

**[Comment 1]”** Introduction

The manuscript didn’t give a clear idea of the central question that the research is intended to answer and its justification.

Make sure you provided a sufficient detail on the magnitude of T2DM among reproductive-age women and the consequences for those affected

The outcome of interest “Knowledge of BRF for T2DM” should have been the central idea in a discussion of why certain factors need more investigation if the problem is to be fully understood. Rather it was "BRF-T2DM" " "behavioral changes -Knowledge" BRF Knowledge - T2DM prevention"…

The argument on non-existence of studies on this topic in Ethiopia was inconsistent with the fact that numerous studies were used to compare and contrast findings later on the discussion section.

The statement on significance of the study doesn't identify the beneficiaries and the benefits of this specific study clearly.’’

**Response:** Thank you very much for pointing this out. We revised the sentence to incorporate the magnitude of T2DM among WRA and its consequences and what this research intend to answer as follows: [In page 4, lines no 58-82], we rearrange the sentence and add, ‘’T2-DM is experiencing rapid global expansion due to several factors, including aging populations, economic development, and urbanization. These changes are all associated with behavioral risk factors (BRFs). The incidence of diabetes also increases gradually during puberty, and it is more prevalent in women, possibly due to hormonal variations and insulin resistance (4). Additionally, it is linked to reproductive issues in individuals of reproductive age, which can be addressed through behavioral changes (7). Around 451 million people over the age of 18 have diabetes, and nearly 22 million women’s live births are affected by the prenatal period (8).

It  also affects fourteen percent of all live births in the Middle East and Africa (9). Preventing diabetes at the population level relies heavily on public awareness and a proper understanding of its risk factors (10).

..

There are multiple factors contributing to the development of T2-DM, categorized as non-modifiable and modifiable risk factors. The modifiable risk factors known as BRFs that can increase the likelihood of developing T2-DM include obesity, physical inactivity, unhealthy eating habits, smoking, excessive alcohol consumption, insomnia, and stress (11, 12). These issues often remain hidden in countries with low socioeconomic status, including our country (13, 14).

BRF significantly predisposes individuals to T2-DM. However, adopting positive lifestyle changes and reducing negative behaviors can reflect an individual's knowledge of these risks (15). Although different studies have assessed knowledge of certain risk factors like sedentary lifestyle, obesity, poor diet, and lack of exercise in diabetes and chronic disease patients, they have overlooked other factors. This study incorporates all possible factors and addresses the limited understanding of knowledge on BRF (10, 15-22). In Ethiopia, research has predominantly concentrated on the general understanding of DM, with limited evidence on the knowledge regarding BRFs (23-25).’’

We also identified the beneficiaries and the benefits of this specific study on page4, line no.83-89

‘’The benefit of this study is improving health outcomes by identifying women's awareness and increasing their knowledge of BRF. It aims to target interventions and inform policy development. The beneficiaries include WRA, health care providers, policy makers, families, and communities.

Therefore, this study assesses WRA's understanding of all potential BRFs for T2-DM and associated factors. This study can also serve as a base for interventions, improve knowledge on healthy lifestyle modifications and T2-DM prevention, and be a reference for scholars.’’

**[Comment 2] ‘’**Materials and Methods

The manuscript contains errors in the design and conduct of research.

For example, the statement on Sample size determination was incorrect given their statistical implications. “q =proportion of people’’

**Response:** Thanks for your comment. We removed the ‘’ q= proportion of people’’,

Some text modification was made to be more coherent on page 5, line 100-103,107,113-125

**[Comment 3] ‘’**A multi-stage sampling strategy doesn’t provide sufficient details on the sampling units and sample allocation and selection procedure.’’

**Response:** Thanks a lot for these comments. We revised the manuscript and elaborate with detail on sampling units and ample selection procedures on page no 5, lines 118-123 as follows: ‘’The computed sample size was proportionally allocated using a multistage sampling method based on the population size in the selected kebeles, after obtaining the number of households from the census. Then every 13th household interval the house was selected until the required sample size was fulfilled. The first house was chosen using the lottery method. If multiple people in a household met the inclusion criteria, only one member was chosen by lottery.’’

**[Comment 4]** ‘’Variable of the study doesn't identify all the variables used in the analysis including Awareness on DM, Knowledge of Type-2DM, Knowledge of symptoms of Type 2-DM, Knowledge relate to prevention of DM and its complication, Behavioral risk Perception for Type 2DM, Adopting healthy life style for Type 2 DM.’’

**Response:** Thanks for your reminder. We added the variable that was identified in the analysis. Page 5, lines no. 124-128.

We considered knowledge of T2-DM, (Symptoms and knowledge related to prevention and complication as general knowledge) and we also included hearing about DM, behavioral risk perception and adopting a healthy life in our analysis but all of them were insignificant

**[Comment 5]** ‘’Data collection was incorrect given their statistical implications “a pre-test of 5 % of the sample population out of the study area” and also described repeatedly. “after sampling technique” and “under a section titled data collection”

Data processing and analysis was incorrect with regard to description of Statistical program used and their purpose. “Epi Data 4.6” Statistical procedures used to modify raw data before analysis. “Combining wrong or uncertain responses in determining level of knowledge of the behavioral risk of T2DM”

Variables used in the analysis “demographic factors” and “the level of knowledge of the behavioral risk of T2DM”

Summary statistics and graphical techniques used in descriptive statistics for each variable.

Methods used for analysis coherently. “Binary logistic regression analysis” “multivariable logistic regression” “Descriptive statistic”

Purpose and reporting format to be followed for the analysis made “Binary logistic regression analysis to identify determinants of behavioural risk factors for T2DM… crude odds ratio (COR) and adjusted odds ratio (AOR) with its respective 95% confidence interval (CI) was used to the number of measurement stations included. Interpret the result. Then, multivariable logistic…”

**Response:** Thank you for the nice reminder. To provide a statistical impression of the text, we readjusted the statements as follows:

“A pre-test of 5 % of the sample population out of the study area” and that was described repeatedly.” Readjusted in the section entitled Data Collection page 6, lines 143- A pretest was conducted with 5% of the sample population who lived outside the study area (Mirab Abaya town)’’

The repetition of the statement about the pretest explanation in sampling was totally removed.

Regarding the description of the statistical programme used and its purpose, It was restated as follows: on page 6, lines 161. ‘’Epi Data 4.6 version was used for data entry, and SPSS version 21 for all statistical analyses.”

This statement was removed totally. ‘’Statistical procedures are used to modify raw data before analysis. “Combining wrong or uncertain responses in determining the level of knowledge of the behavioral risk of T2DM”

Variables used in the analysis are also described as follows, with a restatement on page 7, lines 168: ‘Descriptive statistics was used to summarize and describe the features of the data, and binary logistic regression analysis was applied to assess the association between variables.’

In order to make sentence coherent we restructured as follow on page 7, line168-174: ‘’ …binary logistic regression analysis was applied to assess the association between variables. The variables with p-values less than 0.25 were transferred into multivariate analysis to control the possible confounders. Those variables with a p-value of < 0.05 in the multi-variable analysis were considered to have a significant association with the outcome variable. The Hosmer and Lemeshow test was used to check the assumption tests and models of goodness of fit (P-value = 0.160).”

**[Comment 6]** ‘’Discussion

The discussion was unjustified, inappropriate, and erroneous in many instances in the manuscript. “The knowledge gap in the WRA may worsen the burden of the illness.”

The discussion was also made for each explanatory variable which fails to show association with outcome variable unnecessarily.’’

**Response:** Thank you for your comment. We agreed with you and have completely removed the generalized sentence, 'The knowledge gap in the WRA may worsen the burden of the illness.'

Additionally, the discussion part that explained the variables which failed to show association has been entirely removed from the manuscript.

[**Comment 7**] ‘’Results

The manuscript contain errors in the application, analysis, interpretation, and reporting of statistics. For example

Age was with different measures that requires difference in properties of distribution. “Minimum age of respondents is 15-year-old and maximum age is 49 years” and “mean age of respondents was 27.3 (SD+8.2 years).”

Continuous variable was also reported without measures of variance. “Average family income was 3910.00 Eth birr(--- SD)”

Inconsistent format was used in reporting distribution of variables. 235 (37.7%) were collage and above, “married (57.6%)”, “Half of the participants (312, 50.1%) knew T2DM”

The interpretation of results disregards group sizes for each analysis in many instances in the manuscript. “Source of information (Health workers -116)”, “Have got DM health education (Yes- 159)”

The text report of the regression analysis states a measure of precision (a confidence interval and P values) for each explanatory variable which is unnecessary.

Erroneous interpretation of result was made in some instances. “A household with an average income of more than 5,001 Eth. Birr was also 1.93 times...”

There is no report of whether the variables were assessed for interaction.’’

**Response:** Thanks for your comment. We changed the interpretation of result focused on statistical impression.

Regarding the age was written as follow: The minimum age of respondents is 15 years old, and the maximum age is 49 years old. The mean age of respondents was 27.3 (SD ± 8.2). /page 7 lines no. 192-193./

The continuous variable re writes with SD as follow: the average family income per month was 3910.00 ETH (SD ± 3107.91) under result section./ Page 8 line 196-197/.

The Inconsistent format that was used in reporting distribution of variables also revised accordingly. Number and percent in bracket like this throughout the paper: Concerning educational status, those who were illiterate and able to write as well as read were 27 (4.3%), respectively, and 235 (37.7%) participants were educated in college or above. /page 8, lines no.194-195.

We also revised the inconsistent format used for “Half of the participants (312, 50.1%) knew T2DM”sentence as follow ‘’Approximately half of the participants, 312 (50.1%), were aware that diabetes is incurable,…’’page 8,lines no 214

The interpretation of results disregard group sizes for each analysis was also arranged with proper way: Regarding awareness, almost 568 (91.2%) of study participants had heard about DM, and 264 (42.2%) had gained information from friends or relatives. From the total study participants, 159 (25.4%) had received health education about DM. That means, those who heard about DM were 568 from them the source origin of information from health worker was 116, and those who got health education were 159 from are Page9,table 2.

The text report of the regression analysis that was stated a measure of precision (a confidence interval and P values) for each explanatory variable was removed from all tables and only considered AOR than COR. Hence, we removed the COR column of P-value./page 15,table 8/

Erroneous interpretation of result on the average family income also revised as follow:

‘’Participants with the average family incomes over 3,000 ETH were 1.81 times more likely to possess knowledge than those with incomes below 1,999 ETH. This indicates that those with higher incomes have better access to the necessary knowledge. Overall, the level of knowledge tends to increase with income; as income levels raise, knowledge levels also tend to rise.”

We also assessed the interaction between variable and explained as follow on page 15, lines no. 300-302: A weak correlation was observed among each independent variable. The independent variables do not have a strong linear relationship with each other. In other words, changes in one variable are not strongly associated with changes in another variable.

**[Comment 8]** ‘’Conclusions

Conclusions are presented in inappropriate way. “Nearly 60% of the respondents had no idea about others T2DM risk factors”

The conclusions was also overstated and erroneously discussed possible implications of the results out of the context of data presented in the manuscript. “This might indicate that women

**Response:** Thanks for the critical comment. We think that our conclusion is not perfect and needs improvement. Hence, we deleted unnecessary words and modified them as follows on page 18-19. Line no. 361-370:’’ This community-based cross-sectional study revealed that the overall knowledge about BRF of T2-DM is 47%. A strong statistically significant association was found between average family incomes over 3000 ETH, family history of diabetes, DM in a friend or relative, and diabetes information from friends or relatives, as well as health workers with good knowledge of BRF.

A low knowledge score may indicate a potential knowledge gap. Women, in particular the reproductive age group, need to have an in-depth understanding of BRF to improve their health and prevent the consequences of T2-DM.

Women need equal access to information on T2-DM risk factors, and fostering regular community-based education programs is essential for improving their understanding.”

are ignoring their health.”

The conclusions didn’t provide sufficient details on the association of interest that is “BRF Knowledge” and “Friend /relatives” “Family” “Health worker”’’

**[Comment 9] ‘’**General comments

The language in this manuscript was difficult to understand in many instances and includes.

Grammatical error: for instance ".. is rises.."

Typographical error such as "from he study period"

Unconventional and inappropriate use of abbreviations "RA"

Issues with substantial clinical implications for example " T2DM is evolving"

**Response:** Thanks for the critical comment. We revised accordingly and also removed unconventional abbreviations: RA from the manuscript .
The incidence rises... on Page 3, Line 60 modified. 
‘T2DM is evolving’ change to the correct modification on page 3, line 58.
The text was also modified to be coherent and fluent on page 3.

**[Comment 10]** ‘’In conclusion

These errors are serious enough to question the conclusions.

I recommend authors to read PLOS ONE manuscript submission guidelines and seek editorial help in research report write-up.

Please refer to the revised manuscript.’’

**Response:** Thank you very much for your valuable comment. We modified the point that needed improvement and re wrote the research report based on the submission guideline as much as possible.

**We also entertain the following comments and suggestions regarding the recent remarks in detail.**

**Response to Reviewer `1**

[General Comment]

1.‘’ ‘Is the manuscript technically sound, and do the data support the conclusions?

The manuscript must describe a technically sound piece of scientific research with data Powered by Editorial Manager® and ProduXion Manager® from Aries Systems Corporation that supports the conclusions. Experiments must have been conducted rigorously, with appropriate controls, replication, and sample sizes. The conclusions must be drawn appropriately based on the data presented.’’.’’

Reviewer #1: Yes

Reviewer #4: Partly

Response: Thanks for your valuable comments. We revised the whole conclusions Part based on the data presented in the result and discussion appropriately on page 18-19, number 361-370.

‘’Overall, the knowledge of BRF of T2-DM is 47%. There was a strong statistically significant association between average family incomes greater than 3000 Eth. Birr, having a family history of diabetes, having DM in a friend or relative, sources of information from friends and relatives, and also from health workers.’’

2.Has the statistical analysis been performed appropriately and rigorously?

Response to Reviewer 4

[Comment 1]

Reviewer #1: Yes

Reviewer #4: No

Response: Thanks for your kind reminders. We edited almost all sentences thoroughly with the statistical implications.

3.Have the authors made all data underlying the findings in their manuscript fully available?

Reviewer #1: Yes

Reviewer #4: Yes

Response: Thank you for your comments

4.Is the manuscript presented in an intelligible fashion and written in Standard English?

PLOS ONE does not copyedit accepted manuscripts, so the language in submitted articles must be clear, correct, and unambiguous. Any typographical or grammatical errors should be corrected at revision, so please note any specific errors here.

Reviewer #1: Yes

Reviewer #4: No

Response: Thank you for your suggestion. We revised the entire manuscript's topography, ambiguous articles and grammatical errors accordingly.

Please use the space provided to explain your answers to the questions above. You may also include additional comments for the author, including concerns about dual publication, research ethics, or publication ethics. (Please upload your review as an attachment if it exceeds 20,000 characters)

Reviewer #1: (No Response)

Reviewer #4: Thank you for the opportunity to review this interesting manuscript on women's knowledge about DM type 2 risk factors.

The information is important from the public health point of view, both regional and perhaps world-wide. However, there are some points to be addressed:

1.An English proofing tool should be used, as there are some sentences throughout the text which are not properly constructed.

Response: Thank you for your critical comments. We have revised the entire texts that were improperly constructed so as to make them more appealing to both the reader and the reviewer.

2.Authors should provide some information regarding the representatives of the sample for the Ethiopian female population.

Response: Thank you for your suggestion. We provide information in the material and method section about the population of females in our country on page 4, lines 93-95.

3.The results are a tad difficult to follow and the list of variables is quite long. The logistic regression model is not quite clearly presented.

Response: Thank you for your constructive suggestion. We revised almost those having long variables to make it shorter as much as possible. Almost all long variables in the tables had modified accordingly.(on page 8 table 1, on page 9 table 2, on page 10 table 3, on page 11 table 4, on page 12 table 5, and. on page 13 table 6)

The description in the logistic regression model has been tried to modify on page 13, lines no. 268–287.

4.The Discussion section should be improved - the comparison with other studies should be a tad more detailed, especially with the Ethiopian ones. Also, the results are partly repeated, but not discussed.

Response: Thank you for your suggestion. We revised the Discussion section on page 17-18, line 309-354

5.The conclusions are not quite conclusions - there is a mixture of discussions and general statements.

Response: Thank you for your suggestion. We revised the conclusions on page18-19, line 361-370.’’ This community-based cross-sectional study revealed that the overall knowledge about BRF of T2-DM is 47%. A strong statistically significant association was found between average family incomes over 3000 ETH, family history of diabetes, DM in a friend or relative, and diabetes information from friends or relatives, as well as health workers with good knowledge of BRF.

A low knowledge score may indicate a potential knowledge gap. Women, in particular the reproductive age group, need to have an in-depth understanding of BRF to improve their health and prevent the consequences of T2-DM.

Women need equal access to information on T2-DM risk factors, and fostering regular community-based education programs is essential for improving their understanding.’’

7. PLOS authors have the option to publish the peer review history of their article (what does this mean?). If published, this will include your full peer review and any attached files

If you choose “no”, your identity will remain anonymous but your review may still be made public.

Do you want your identity to be public for this peer review? For information about this choice, including consent withdrawal, please see our Privacy Policy.

Reviewer #1: No

Reviewer #4: No

Response: Thank you for your suggestion
