# Supplementary material for: Knowledge of behavioral risk factors for type 2 diabetes mellitus and its associated factors among women of reproductive age
Source: PLoS One. 2025 Feb 13;20(2):e0275700. doi: 10.1371/journal.pone.0275700 (PMC11824971; doi:10.1371/journal.pone.0275700)
Supplement: Attachment — Submitted filename: Response to Reviewers of PLOSE.docx [file pone.0275700.s003.docx]

**RESPONSE TO REVIEWER**

# Cover Letter

Dear Editor,

We genuinely appreciate you and the reviewers for taking the time to review our paper and provide valuable comments. Your insightful feedback has significantly contributed to the improvements in the current version. We have carefully considered each comment and have made every effort to address them. We hope that the revised manuscript meets your high standards. We also welcome any further constructive comments. Below, we provide point-by-point responses. All modifications in the manuscript have been highlighted in blue on a white background.

Sincerely,

Tinsae Seyoum (PI)

[tswy2006@gmail.com](mailto:tswy2006@gmail.com)

+251913260781

Lecturer, Department of Public Health

Arba Minch College of Health Sciences

Contents

Cover Letter	1

[Response to previous comments:	3](#_Toc185135669)

[Response to general comments	3](#_Toc185135670)

[Response to Reviewer 1	3](#_Toc185135671)

[Response to Reviewer 2	3](#_Toc185135672)

[Response to Reviewer 3	9](#_Toc185135673)

[Response for recent comments	19](#_Toc185135674)

[Response to Reviewer `1 and 4	19](#_Toc185135675)

# The Previous reviewer general comments

**[General comments]** ‘’When submitting your revision, we need you to address these additional requirements.

1. Please ensure that your manuscript meets PLOS ONE's style requirements, including those for file naming. The PLOS ONE style templates can be found at
   [https://journals.plos.org/plosone/s/file?id=wjVg/PLOSOne_formatting_sample_main_body. pdf](https://journals.plos.org/plosone/s/file?id=wjVg/PLOSOne_formatting_sample_main_body.%20pdf) and [https://journals.plos.org/plosone/s/file?id=ba62/PLOSOne_formatting_sample_title_authors_affiliations.pdf](https://journals.plos.org/plosone/s/file?id=ba62/PLOSOne_formatting_sample_title_authors_affiliations.pdf#inbox/_blank)

2. You indicated that you had ethical approval for your study. In your Methods section, please ensure you have also stated whether you obtained consent from parents or guardians of the minors (participants below the age of 18 years) included in the study or whether the research ethics committee or IRB specifically waived the need for their consent.

3. When completing the data availability statement of the submission form, you indicated that you will make your data available on acceptance. We strongly recommend all authors decide on a data sharing plan before acceptance, as the process can be lengthy and hold up publication timelines. Please note that, though access restrictions are acceptable now, your entire data will need to be made freely accessible if your manuscript is accepted for publication. This policy applies to all data except where public deposition would breach compliance with the protocol approved by your research ethics board. If you are unable to adhere to our open data policy, please kindly revise your statement to explain your reasoning and we will seek the editor's input on an exemption. Please be assured that, once you have provided your new statement, the assessment of your exemption will not hold up the peer review process.

4. Please include your tables as part of your main manuscript and remove the individual files. Please note that supplementary tables (should remain/ be uploaded) as separate "supporting information" files

# Response to previous reviewer comments:

## Response to general comments

Response to General comment 1: Thank you for your comment. We have revised the article title, the symbol legend, and the corresponding author email initials in parentheses based on the PLOS ONE style templates.

Response to General comment 2: Thank you for your valuable comment. We have indicated that the ethical approval for our study obtained consent from parents and guardians of the minors (participants below the age of 18 years) included in the study on page 7, lines 183-187.

Response to General comment 3: Thank you for your comment. We have declared that our entire data will be made freely accessible if this manuscript is accepted for publication.

Response to General comment 4: Thank you for your nice comment. We have incorporated the tables into the main manuscript and removed the individual file as per the comments.

## Response to Reviewer 1

**[General Comment]** ‘’This is an important article on knowledge of behavioral risk factors of **Diabetes** among reproductive age group women in Ethiopia. However, the manuscript needs editing throughout in terms of English language, typos, abbreviations and the result section needs to be articulated in a proper fashion. Thanks’’

**Response 1:** Thank you for your valuable comments. We have revised the English language, corrected typos, and clarified abbreviations. We have also articulated the results section properly throughout the entire paper, from the abstract to the conclusion.

## Response to Reviewer 2

**[General Comment 2]** ‘’I was kindly asked to review the manuscript of Seyoum T et al. I thank the authors for their work, as the result is interesting and achieved through a very well described and reproducible methodological rigor. I believe the article may also be of interest to readers of the journal, but before publication, it should, in my opinion, be fixed in form, following the suggestions given below.’’

**Response.2:** Thank you very much for your comments that helped us improves this manuscript.

**[Comment 2.1] ‘’**Editing for English language is recommended. Line numbers in the manuscript file are missing.’’

**Response 2.1:** Thanks for your kind reminders. We have edited almost all sentences thoroughly and provided line numbers in the whole manuscript file.

**[Comment 2.2]** Title:

‘’I suggest deleting “2022” at the end of the title and using “…in Arba Minch, Gamo zone, Southern Ethiopia” here and everywhere into the main text, abstract included.’’

**Response 2.2:** Thanks. We edited the title page, abstract and the main texts by removing ‘’2022’’ and’’ in Arba Minch, Gamo zone, South Ethiopia’’ except from the tables and figures.

**The revised Title**: Knowledge of behavioral risk factors for type 2 ddiabetes mellitus and its associated factors among reproductive-age women on page 1, line1-3

Abstract: **Objectives**, To assess knowledge of the behavioral risks of type 2 diabetes mellitus and its associated factors among women of reproductive age./page 2, line 22-23/

**[Comment 2.3] ‘’**Corresponding author:

Please, report on the same line the corresponding author and the email address’’

**Response 2.3:** Thanks for your kind reminders. Thank you for your kind reminders. We have made the necessary edits accordingly.

*= Corresponding author: Email [address,tswy2006@gmail.com](mailto:address,tswy2006@gmail.com) (T.S) on page no 1, line 12

**[Comment 2.4] ‘’**Authorship:

Please, verify with the Editor that, beside a first joint authorship, another equal contribution is admitted.

Please, delete the #aArba Minch, South Nation Nationality Region, Ethiopia, as affiliations report this information.’’

**Response 2.4:** Thank you for your valuable comment. We have verified the equal contribution of the authors. We removed the symbol '#@' and replaced it with '¶' and '&' for equal contribution, based on the PLOS ONE guidelines. Additionally, we removed the sentence '#a, Arba Minch, South Nation Nationality Region, Ethiopia.' from the cover page (Page 1).

**[Comment 2.5]** ‘’Abstract, page 2:

Define abbreviations upon first appearance in the text. For example, the abbreviations AOR,

BRF and DM are cited, but not defined.

Please, verify the order of words at the end of the Objective sentence and as suggested previously.’’

**Response 2.5:** Thank you for your nice suggestion. We revised the abbreviations on the abstract page 2, lines 30, 31, 32, 34, and 39 to “Adjusted Odd Ratio, Behavioral Risk Factors, and Diabetes Mellitus.” The objective was also amended according to your suggestions and comments by removing ‘2022’ and ‘in Arba Minch town, South Ethiopia.’

**[Comment 2.6]** ‘’Introduction:

Line 2, please add a space between diabetes and (T2DM)

Line 28, please remove the space at the beginning of the sentence.

Line 32, please define the abbreviation “RA” before using it

Line 35-38, please evaluate to move this paragraph before the sentence “As a result, …” at line

31 or in the discussions section.’’

**Response 2.6:** Thank you for your valuable comment. We have made the necessary revisions accordingly. The paragraph stating 'Knowledge of BRF is also a prerequisite for preventing diabetes in reproductive age groups' has been moved to the discussion section.

**[Comment 2.7]** ‘’Results:

I suggest renaming sub-headings or numbering the entire section as “5.RESULTS” and the ‘’

**Response 2.7:** Thank you for your valuable comment. We have renamed the subheading section and removed the numbering styles as per your suggestion.

**[Comment 2.8]** ‘’Materials and methods:

Please, define the study design: qualitative, quantitative or mix-method?

Please, correct the study period: from February 20, 2022 to March 22, 2022, not February20/2022.

Please, correct “women of reproductive age”. In my personal opinion, a woman of reproductive age is 46 years old, at maximum. I strongly recommend defining the fertile age among inclusion criteria, excluding from the study women of more than 46 years old and revising the manuscript accordingly.

**Response 2.8:** Thank you for your eminent comments and we revised accordingly as follow: Thank you for your eminent comments. We have made the following revisions accordingly:

- We defined the study design by adding "quantitative" on page 4, line 90.
- We corrected the study period to February 20, 2022, to March 22, 2022 (Page 4, line 91).
- We Changed the text to "women of reproductive age" throughout the manuscript, including page 1, lines 2-3; WRA on page 2, lines 23, 31, and 43, and in the keywords.
- Regarding the participants' age, we used data from the World Health Organization (WHO) and the Ethiopian Demographic Health Survey (EDHS) to define a woman of reproductive age as being between 15 and 49 years old. All analyses were conducted for ages 15 to 49. Considering age up to 46 would affect the entire data and results.
- Unfortunately, we did not consider the "parity" variable in this study. We have recommended addressing this in future research in the recommendation section (Page 19, lines 389-390).

What about parity? It should be of interest to analyses wherever parity > 0 may be associated with higher or lower consciousness.’’

**[Comment 2. 9]** ‘’Discussion and Conclusion:

Section are numbered. Either all sections are numbered, or no section is numbered, your choice!’’

**Response 2.9:** Thank you for your valuable comment. We have revised the manuscript by removing numbers from all sections as per your suggestion.

**[Comment 2.10] ‘’**References:

Please, use proper formatting and the same font.

What does it mean “uncategorized references”?’’

**Response 2.10:** Thank you for your valuable suggestion. We have revised the fonts in the references to ensure consistency at all levels and adjusted the formats using EndNote accordingly. Additionally, the uncategorized part has been completely removed.

### Response to Reviewer 3

**[Comment 3.1]”** Introduction

The manuscript didn’t give a clear idea of the central question that the research is intended to answer and its justification.

Make sure you provided a sufficient detail on the magnitude of T2DM among reproductive-age women and the consequences for those affected

The outcome of interest “Knowledge of BRF for T2DM” should have been the central idea in a discussion of why certain factors need more investigation if the problem is to be fully understood. Rather it was "BRF-T2DM" " "behavioral changes -Knowledge" BRF Knowledge - T2DM prevention"…

The argument on non-existence of studies on this topic in Ethiopia was inconsistent with the fact that numerous studies were used to compare and contrast findings later on the discussion section.

The statement on significance of the study doesn't identify the beneficiaries and the benefits of this specific study clearly.’’

**Response 3.1:** Thank you very much for pointing this out. We revised the sentence to incorporate the magnitude of T2DM among WRA and its consequences and what this research intend to answer as follows: [In page 3-4, lines no 58-85], we rearrange the sentence and add, ‘’T2-DM is experiencing rapid global expansion due to several factors, including aging populations, economic development, and urbanization. These changes are all associated with BRFs. The incidence of diabetes also increases gradually during puberty, and it is more prevalent in women, possibly due to hormonal variations and insulin resistance (4). Additionally, it is linked to reproductive issues in individuals of reproductive age, which can be addressed through behavioral changes (7). Around 451 million people over the age of 18 have diabetes, and nearly 22 million women’s live births are affected by the prenatal period (8).

It  also affects fourteen percent of all live births in the Middle East and Africa (9). Preventing diabetes at the population level relies heavily on public awareness and a proper understanding of its risk factors (10).

There are multiple factors contributing to the development of T2-DM, categorized as non-modifiable and modifiable risk factors. The modifiable risk factors known as BRFs that can increase the likelihood of developing T2-DM include obesity, physical inactivity, unhealthy eating habits, smoking, excessive alcohol consumption, insomnia, and stress (11, 12). These issues often remain hidden in countries with low socioeconomic status, including our country (13, 14).

BRF significantly predisposes individuals to T2-DM. However, adopting positive lifestyle changes and reducing negative behaviors can reflect an individual's knowledge of these risks (15). Although different studies have assessed knowledge of certain risk factors like sedentary lifestyle, obesity, poor diet, and lack of exercise in diabetes and chronic disease patients, they have overlooked other factors. This study incorporates all possible factors and addresses the limited understanding of knowledge on BRF (10, 15-22). In Ethiopia, research has predominantly concentrated on the general understanding of DM, with limited evidence on the knowledge regarding BRFs (23-25).’’

We also identified the beneficiaries and the benefits of this specific study on page4, line no.83-89

‘’The benefit of this study is improving health outcomes by identifying women's awareness and increasing their knowledge of BRF. It aims to target interventions and inform policy development. The beneficiaries include WRA, health care providers, policy makers, families, and communities.

Therefore, this study assesses WRA's understanding of all potential BRFs for T2-DM and associated factors. This study can also serve as a base for interventions, improve knowledge on healthy lifestyle modifications and T2-DM prevention, and be a reference for scholars.’’

**[Comment 3.2] ‘’**Materials and Methods

The manuscript contains errors in the design and conduct of research.

For example, the statement on Sample size determination was incorrect given their statistical implications. “q =proportion of people’’

**Response 3.2:** Thanks for your comment. We removed the ‘’ q= proportion of people’’,

Some text modification was made to be more coherent on page 5, line 100-103,104,111-113,116-128

**[Comment 3.3] ‘’**A multi-stage sampling strategy doesn’t provide sufficient details on the sampling units and sample allocation and selection procedure.’’

**Response 3.3:** Thanks a lot for these comments. We revised the manuscript and elaborate with detail on sampling units and ample selection procedures on page no 5, lines 118-123 as follows: ‘The sample size was determined using a multistage sampling method, which allocated participants proportionally based on the population size of the selected kebeles. After obtaining the number of households from the census, every 13th household was selected until the required sample size was achieved. The first house was selected using a lottery method. If multiple members of a household met the inclusion criteria, only one individual was chosen through the lottery.’’

**[Comment 3.4]** ‘’Variable of the study doesn't identify all the variables used in the analysis including Awareness on DM, Knowledge of Type-2DM, Knowledge of symptoms of Type 2-DM, Knowledge relate to prevention of DM and its complication, Behavioral risk Perception for Type 2DM, Adopting healthy life style for Type 2 DM.’’

**Response 3.4:** Thanks for your reminder. We added the variable that was identified in the analysis. Page 5, lines no. 124-128.

In this study, knowledge of BRF is the dependent variable. The independent variables include general knowledge of T2-DM (such as its meaning, symptoms, prevention, and control), behavioral risk perception, leading a healthy lifestyle, socio-demographic factors, and other relevant factors encompass personal history of diabetes, family history of diabetes, exposure to diabetes health education, awareness of diabetes, and sources of information.

**[Comment 3.5]** ‘’Data collection was incorrect given their statistical implications “a pre-test of 5 % of the sample population out of the study area” and also described repeatedly. “after sampling technique” and “under a section titled data collection”

Data processing and analysis was incorrect with regard to description of Statistical program used and their purpose. “Epi Data 4.6” Statistical procedures used to modify raw data before analysis. “Combining wrong or uncertain responses in determining level of knowledge of the behavioral risk of T2DM”

Variables used in the analysis “demographic factors” and “the level of knowledge of the behavioral risk of T2DM”

Summary statistics and graphical techniques used in descriptive statistics for each variable.

Methods used for analysis coherently. “Binary logistic regression analysis” “multivariable logistic regression” “Descriptive statistic”

Purpose and reporting format to be followed for the analysis made “Binary logistic regression analysis to identify determinants of behavioural risk factors for T2DM… crude odds ratio (COR) and adjusted odds ratio (AOR) with its respective 95% confidence interval (CI) was used to the number of measurement stations included. Interpret the result. Then, multivariable logistic…”

**Response 3.5:** Thank you for the nice reminder. To provide a statistical impression of the text, we readjusted the statements as follows:

“A pre-test of 5 % of the sample population out of the study area” and that was described repeatedly.” Readjusted in the section entitled Data Collection page 6, lines 143-144- ‘A pretest was conducted with 5% of the sample population who lived outside the study area (Mirab Abaya town).’’

The repetition of the statement about the pretest explanation in sampling was totally removed.

Regarding the description of the statistical programme used and its purpose, It was restated as follows: on page 6, lines 161. ‘’Data entry was performed using Epi Data version 4.6, while all statistical analyses were conducted with SPSS version 21.”

This statement was removed totally. ‘’Statistical procedures are used to modify raw data before analysis. “Combining wrong or uncertain responses in determining the level of knowledge of the behavioral risk of T2DM”

Variables used in the analysis are also described as follows, with a restatement on page 7, lines 169-170: ‘Descriptive statistics was used to summarize and describe the features of the data, and binary logistic regression analysis was applied to assess the association between variables.’

In order to make sentence coherent we restructured as follow on page 7, line169-175: ‘’ …binary logistic regression analysis was applied to assess the association between variables. The variables with p-values less than 0.25 were transferred into multivariate analysis to control the possible confounders. Those variables with a p-value of < 0.05 in the multi-variable analysis were considered to have a significant association with the outcome variable. The Hosmer and Lemeshow test was used to check the assumption tests and models of goodness of fit (P-value = 0.160).”

**[Comment 3.6]** ‘’Discussion

The discussion was unjustified, inappropriate, and erroneous in many instances in the manuscript. “The knowledge gap in the WRA may worsen the burden of the illness.”

The discussion was also made for each explanatory variable which fails to show association with outcome variable unnecessarily.’’

**Response 3.6:** Thank you for your comment on the sentence "The knowledge gap in the WRA may worsen the burden of the illness." This sentence expresses our understanding of the study results. If women of reproductive age do not have sufficient knowledge about the behavioral risk factors of T2-DM, it may lead to the illness. Improving their knowledge is essential to help reduce the burden of it in this group of age. However, upon review, we agreed with your suggestion and have completely removed the generalized sentence.

Additionally, the discussion part that explained the variables which failed to show association has been entirely removed from the manuscript.

[**Comment 3.7**] ‘’Results

The manuscript contain errors in the application, analysis, interpretation, and reporting of statistics. For example

Age was with different measures that requires difference in properties of distribution. “Minimum age of respondents is 15-year-old and maximum age is 49 years” and “mean age of respondents was 27.3 (SD+8.2 years).”

Continuous variable was also reported without measures of variance. “Average family income was 3910.00 Eth birr(--- SD)”

Inconsistent format was used in reporting distribution of variables. 235 (37.7%) were collage and above, “married (57.6%)”, “Half of the participants (312, 50.1%) knew T2DM”

The interpretation of results disregards group sizes for each analysis in many instances in the manuscript. “Source of information (Health workers -116)”, “Have got DM health education (Yes- 159)”

The text report of the regression analysis states a measure of precision (a confidence interval and P values) for each explanatory variable which is unnecessary.

Erroneous interpretation of result was made in some instances. “A household with an average income of more than 5,001 Eth. Birr was also 1.93 times...”

There is no report of whether the variables were assessed for interaction.’’

**Response 3.7:** Thank you for your valuable comments. We have made the following revisions accordingly:

- We changed the interpretation of results to focus on statistical impressions.
- Regarding age, we wrote: "The minimum age of respondents is 15 years old, and the maximum age is 49 years old. The mean age of respondents was 27.3 (SD ± 8.2)" (Page 7, lines 194-195).
- For the continuous variable, we rewrote it with SD as follows: "The average family income per month was 3910.00 ETH (SD ± 3107.91)" under the results section (Page 8, lines 198-199).
- Revised the inconsistent format used in reporting the distribution of variables. Number and percent are now in brackets throughout the paper: "The study found that 27 (4.3%) individuals were illiterate and able to write and read, while 235 (37.7%) of participants had education at the college level and above " (Page 8, lines 196-198).
- We revised the statement and inconsistent format used for "Half of the participants (312, 50.1%) knew T2DM" to: "Approximately half of the participants, 312 (50.1%), were aware that diabetes is incurable" (Page 8, lines 216-217).
- We arranged the interpretation of results with proper group sizes for each analysis: "The study revealed that 568 (91.2%) participants were aware of diabetes, with 264 (42.2%) having information from their friends or relatives, 159 (25.4%) receiving health education about T2-DM, and 192 (31%) having their friends or relatives with diabetes; besides this, 134 (21.5%) had diabetes in their families (Table 2).Page 9, line 203-206
- We removed the COR column of P-value from all tables and only considered AOR in the text report of the regression analysis (Page 15, Table 8).
- We also revised the erroneous interpretation of results on the average family income as follows: "Participants with average family incomes over 3,000 ETH were 1.81 times more likely to possess knowledge than those with incomes below 1,999 ETH. This indicates that those with higher incomes have better access to the necessary knowledge. Overall, the level of knowledge tends to increase with income; as income levels rise, knowledge levels also tend to rise. ‘’ page 17, line 328-334
- Assessed the interaction between variables and explained as follows: "A weak correlation was observed among each independent variable. The independent variables do not have a strong linear relationship with each other. In other words, changes in one variable are not strongly associated with changes in another variable" (Page 15, lines 302-304)

**[Comment 3.8]** ‘’Conclusions

Conclusions are presented in inappropriate way. “Nearly 60% of the respondents had no idea about others T2DM risk factors”

The conclusions was also overstated and erroneously discussed possible implications of the results out of the context of data presented in the manuscript. “This might indicate that women

**Response 3.8:** Thank you for your critical comment. We acknowledge that our conclusion is not perfect and needs improvement. Therefore, we have deleted unnecessary words and modified it as follows on page 18-19, lines 363-372:

"This community-based cross-sectional study revealed that the overall knowledge about Behavioral Risk Factors (BRF) of Type 2 Diabetes Mellitus (T2-DM) is 47%. A strong statistically significant association was found between average family incomes over 3000 ETH, family history of diabetes, diabetes in a friend or relative, and diabetes information from friends or relatives, as well as health workers with good knowledge of BRF.

A low knowledge score may indicate a potential knowledge gap. Women, particularly those in the reproductive age group, need to have an in-depth understanding of BRF to improve their health by minimizing the risk of T2-DM and its consequences. Women need more access to information on T2-DM risk factors. Additionally, fostering regular community-based health education programs is essential for improving their understanding."

are ignoring their health.”

The conclusions didn’t provide sufficient details on the association of interest that is “BRF Knowledge” and “Friend /relatives” “Family” “Health worker”’’

**[Comment 3.9] ‘’**General comments

The language in this manuscript was difficult to understand in many instances and includes.

Grammatical error: for instance ".. is rises.."

Typographical error such as "from he study period"

Unconventional and inappropriate use of abbreviations "RA"

Issues with substantial clinical implications for example "T2DM is evolving"

**Response 3.9:** Thank you for the critical comment. We have revised accordingly and removed the unconventional abbreviation "RA" from the manuscript.

- The incidence rises... on Page 3, Line 60 has been modified.
- "T2DM is evolving" has been changed to the correct modification on Page 3, Line 58.
- The text on Page 3 has also been modified to be more coherent and fluent.

**[Comment 3.10]** ‘’In conclusion

These errors are serious enough to question the conclusions.

I recommend authors to read PLOS ONE manuscript submission guidelines and seek editorial help in research report write-up.

Please refer to the revised manuscript.’’

**Response 3.10:** Thank you very much for your valuable comment. We have modified the points that needed improvement and rewritten the manuscript based on the submission guidelines as much as possible.

**We also welcomed a detailed comments and suggestions regarding the recent remarks**

# Response for recent comments

### Response to Reviewer `1 and 4

[General Comment]

1. **‘’ ‘Is the manuscript technically sound, and do the data support the conclusions?**

The manuscript must describe a technically sound piece of scientific research with data Powered by Editorial Manager® and Produ Xion Manager® from Aries Systems Corporation that supports the conclusions. Experiments must have been conducted rigorously, with appropriate controls, replication, and sample sizes. The conclusions must be drawn appropriately based on the data presented.’’.’’

Reviewer #1: Yes

Reviewer #4: Partly

Response: Thank you for your valuable comments. We have revised the entire conclusions section based on the data presented in the results and discussion, specifically on page 18-19, lines 363-372:

"This community-based cross-sectional study revealed that the overall knowledge about Behavioral Risk Factors (BRF) of Type 2 Diabetes Mellitus (T2-DM) is 47%. A strong statistically significant association was found between average family incomes over 3000 ETH, family history of diabetes, diabetes in a friend or relative, and diabetes information from friends or relatives, as well as health workers with good knowledge of BRF."

**Response to Reviewer 4**

2. **Has the statistical analysis been performed appropriately and rigorously?**

[Comment 1]

Reviewer #1: Yes

Reviewer #4: No

Response: Thank you for your kind reminders. We have thoroughly edited almost all sentences to include the statistical implications.

3. **Have the authors made all data underlying the findings in their manuscript fully available?**

Reviewer #1: Yes

Reviewer #4: Yes

Response: Thank you for your comments.

4. **Is the manuscript presented in an intelligible fashion and written in Standard English?**

PLOS ONE does not copyedit accepted manuscripts, so the language in submitted articles must be clear, correct, and unambiguous. Any typographical or grammatical errors should be corrected at revision, so please note any specific errors here.

Reviewer #1: Yes

Reviewer #4: No

Response: Thank you for your suggestion. We have revised the entire manuscript's topography, clarified ambiguous articles, and corrected grammatical errors accordingly.

Please use the space provided to explain your answers to the questions above. You may also include additional comments for the author, including concerns about dual publication, research ethics, or publication ethics. (Please upload your review as an attachment if it exceeds 20,000 characters)

Reviewer #1: (No Response)

Reviewer #4: Thank you for the opportunity to review this interesting manuscript on women's knowledge about DM type 2 risk factors.

The information is important from the public health point of view, both regional and perhaps world-wide. However, there are some points to be addressed:

Response: Thank you for your suggestion and critical view on this manuscript. We appreciate your feedback and have made the necessary revisions accordingly.

5. **An English proofing tool should be used, as there are some sentences throughout the text which are not properly constructed.**

Response: Thank you for your critical comments. We have revised the entire text that was improperly constructed to make it more appealing to both the reader and the reviewer.

6. **Authors should provide some information regarding the representatives of the sample for the Ethiopian female population.**

Response: Thank you for your suggestion. We provide information in the material and method section about the population of females in our country on page 4, lines 93-95.

7. **The results are a tad difficult to follow and the list of variables is quite long. The logistic regression model is not quite clearly presented.**

Response: Thank you for your constructive suggestion. We revised almost those having long variables to make it shorter as much as possible. Almost all long variables in the tables had modified accordingly.(on page 8 table 1, on page 9 table 2, on page 10 table 3, on page 11 table 4, on page 12 table 5, on page 13 table 6 and on page 14 table 7)

The description in the logistic regression model has been tried to modify on page 15, lines no. 280–304.

8. **The Discussion section should be improved - the comparison with other studies should be a tad more detailed, especially with the Ethiopian ones. Also, the results are partly repeated, but not discussed.**

Response: Thank you for your suggestion. We revised the Discussion section on page 17-18, line 311-356

9. **The conclusions are not quite conclusions - there is a mixture of discussions and general statements.**

Response: Thank you for your suggestion. We revised the conclusions on page18-19, line 363-372.’’ This community-based cross-sectional study revealed that the overall knowledge about Behavioral Risk Factors (BRF) of Type 2 Diabetes Mellitus (T2-DM) is 47%. A strong statistically significant association was found between average family incomes over 3000 ETH, family history of diabetes, diabetes in a friend or relative, and diabetes information from friends or relatives, as well as health workers with good knowledge of BRF.

A low knowledge score may indicate a potential knowledge gap. Women, particularly those in the reproductive age group, need to have an in-depth understanding of BRF to improve their health by minimizing the risk of T2-DM and its consequences. Women need more access to information on T2-DM risk factors. Additionally, fostering regular community-based health education programs is essential for improving their understanding.’’

.’’

10. **PLOS authors have the option to publish the peer review history of their article (what does this mean?). If published, this will include your full peer review and any attached files**

If you choose “no”, your identity will remain anonymous but your review may still be made public.

Do you want your identity to be public for this peer review? For information about this choice, including consent withdrawal, please see our Privacy Policy.

Reviewer #1: No

Reviewer #4: No

Response: Thank you for your view. You're welcome! If you have any more questions and comments.
